# Reading and Writing Skills in Adolescents With Autism Spectrum Disorder Without Intellectual Disability

**DOI:** 10.3389/fpsyg.2021.646849

**Published:** 2021-07-19

**Authors:** Inmaculada Baixauli, Belen Rosello, Carmen Berenguer, Montserrat Téllez de Meneses, Ana Miranda

**Affiliations:** ^1^Occupational Sciences, Speech Language Therapy, Developmental and Educational Psychology Department, Catholic University of Valencia, Campus Capacitas, Valencia, Spain; ^2^Developmental and Educational Psychology, University of Valencia, Valencia, Spain; ^3^Neuropediatrics Section, Hospital la Fe de Valencia, Valencia, Spain

**Keywords:** autism, reading, writing, academic achievement, secondary school

## Abstract

The purpose of this study is to extend the knowledge about academic achievement in individuals with autism spectrum disorder (ASD). To this end, first, we analyzed differences in a wide range of reading and writing skills in adolescents with ASD without intellectual disability (ASD-WID) and adolescents with typical development (TD). Second, these two groups were compared on academic outcomes in core subjects and indicators of successful transition to secondary school. Third, the potential contribution of literacy skills to academic outcomes was examined in the two groups. Participants were 56 adolescents between 12 and 14 years old, 30 with ASD-WID and 26 with TD. Results showed no significant differences between the two groups on measures of reading fluency or literal and inferential comprehension. However, the performance of the group with ASD was significantly lower on reading comprehension processes that assess cognitive flexibility. Regarding their written expression skills, significant differences were observed between the group with ASD and the group with TD on most of the indicators analyzed as: productivity, lexical diversity, and overall coherence (resolution component). In addition, findings showed that the deficits in reading and writing observed in the adolescents with ASD significantly affected their academic achievement, which was lower than that of their peers with TD and below what would be expected based on their intellectual capacity. Moreover, their families’ perceptions of the transition to high school reflected worse adjustment and lower self-esteem, confidence, and motivation.

## Introduction

Autism spectrum disorder (ASD) is a neurodevelopmental condition characterized by persistent difficulties in communication and social interactions, along with restricted interests and the presence of repetitive behaviors (American Psychiatric Association, 2013). A key factor in the development of people with ASD is academic achievement, which undoubtedly contributes to access to employment opportunities and independent living. The inclusion of students with ASD in regular classrooms is a growing reality, and so it is necessary to analyze the factors that influence their educational performance.

Despite the relevance of this topic, research on the academic profile of students with ASD is limited, particularly in the adolescent stage or during the transition to secondary school. This is a complex period when important developmental changes occur, and it is characterized by greater academic and social demands. The inherent characteristics of ASD, such as difficulties in social communication, resistance to change, sensory hypersensitivity, or intolerance to uncertainty, make these students a particularly vulnerable population in this developmental period. In fact, anxiety, social pressure, and bullying have been found to be significant challenges for adolescents with ASD (Nuske et al., 2018), who report negative experiences in this academic transition phase (Makin et al., 2017). Therefore, identifying the variables that influence school performance and contribute to successful transition to secondary school should be a major research objective.

To date, studies that have attempted to analyze the academic performance of students with ASD have generally referred a variable performance, which is consistent with the disorder’s heterogeneity (Keen et al., 2016). To account for this variability, studies have tried to establish different performance profiles in children and adolescents with diverse cognitive abilities. Thus, Wei et al. (2014) identified four distinct profiles based on five measures of academic achievement (word identification, rapid letter naming, passage comprehension, applied problems, and calculation). These profiles were the following: higher-achieving (39%, who have scored around the national average on most of the measures), hyperlexia (9%, who do well on decoding but with poor comprehension), hypercalculia (20%, with scores close to the national average on calculation skills, but significantly below average on the other four dimensions), and lower-achieving (32%, children who scored about two standard deviations below the mean on all five indicators). The higher-achieving and hyperlexia subgroups had significantly better functional cognitive skills and came from higher socioeconomic backgrounds.

Following a similar approach, Chen et al. (2019), in a sample 7–14 years old participants with ASD with varied intelligence levels, could determine two distinct groups, low-achievement ASD and high-achievement ASD, based on their performance on four measures taken from the WIAT-II (Weschler, 2001): numerical operations, mathematical reasoning, word reading, and reading comprehension. These differences were especially pronounced in the area of mathematics. As in the study by Wei et al. (2014), the low-achievement subgroup scores were consistent with the intellectual capacity and other cognitive processes, such as working memory.

However, despite the expected relationship between cognitive ability and academic performance, other studies have identified discrepancies in this regard. Jones et al. (2009) found that approximately 70% of adolescents with ASD from 14 to 16 years old with different cognitive levels had a significant divergence between intellectual ability and one or more achievement domains. They described four subgroups in which either word reading (“reading peak” and “reading dip”) or arithmetic (“arithmetic peak” and “arithmetic dip”) was higher or lower than the WASI Full Scale Intelligence Quotient (IQ; Wechsler, 1999). Estes et al. (2011) identified these same differences in a sample of 30 children with ASD without intellectual disabilities. In 60% of them, a significantly lower achievement was found than what was predicted by their cognitive ability, in at least one of the domains analyzed (spelling, word reading, or basic number skills).

In conclusion, students with ASD present an irregular performance profile where intelligence plays an important role, although the research is not consistent in this regard. However, it is clear that many children and adolescents with ASD perform below what would be expected based on their intellectual capacity. Therefore, it is necessary to determine the areas of vulnerability and the factors involved in this low performance, in order to plan more appropriate and effective interventions. Certainly, in the school context, reading and writing are crucial instrumental skills and the basis for success in different curricular areas, and they have important implications throughout life, not only academically, but also socially and occupationally.

### Reading Difficulties in ASD

Reading is a complex skill involving the orchestration of different components. From an overall perspective, two major processes may be distinguished as: decoding and comprehension. Decoding refers to the transformation of written words (graphemes) into phonological representations. Comprehension processes refer to the extraction of meaning from the written text. Specifically, and according to one of the most known models of reading, the Simple View of Reading Model (Hoover and Gough, 1990), reading comprehension is the product of decoding skills and linguistic comprehension.

Many studies have found that people with ASD without intellectual disabilities (ASD-WID) have strengths in decoding skills, compared to their reading comprehension performance. Thus, in general, students with ASD-WID seem to master mechanisms involved in automatic word recognition, in contrast to their reading comprehension performance (Jones et al., 2009; Norbury and Nation, 2011; Solari et al., 2017), which is below expectations for their chronological age or reading accuracy level (Brown et al., 2013). To explain this discrepancy, good memory skills have been cited, as well as phonological and visual processing skills, which would contribute to adequate recognition of the written word.

As in the case of academic performance, the variability in reading skills has led studies to identify reading profiles in samples of children and adolescents with ASD. In a longitudinal and retrospective study, Åsberg et al. (2019) were able to determine three subgroups. A first subgroup, with “low reading performance,” which was the most frequent profile (approximately 50% of the participants), had below-average scores on both word reading and text comprehension. A second subgroup, made up of “skilled readers,” performed above average on both reading processes. Finally, the third subgroup was the least common (20%), and it was composed of “hyperlexical/low understanding” participants. Following a similar approach, namely latent profile analysis, McIntyre et al. (2017) found four different profiles in a sample of students with high-functioning autism from 8 to 16 years old: (1) readers with overall disturbance (32.2%), i.e., alterations in the decoding and comprehension processes; (2) readers with severe overall disturbance (14.1%); (3) readers with comprehension problems (20.6%); and (4) average readers (32.1%). In other words, approximately 70% of the participants experienced reading impairments, and of them, the majority had comprehension problems. Other studies using a cluster analysis methodology have also found lower reading comprehension scores than word reading scores, even in high-achieving subgroups of students with ASD (Chen et al., 2019).

To determine the factors moderating reading comprehension performance in students with ASD, Brown et al. (2013) conducted a meta-analysis of 36 studies comparing ASD and control groups. The strongest individual predictors of reading comprehension were semantic knowledge and decoding skills. The study results highlight the contribution of oral language to reading comprehension, as well as the content of the texts, because individuals with ASD were significantly worse at comprehending highly social texts than less social texts.

In sum, decoding skills, language level, and text characteristics and content are variables to take into account when analyzing the reading comprehension difficulties of students with ASD. Other factors to consider are the development of comprehension strategies during reading (Williamson et al., 2012) or the type of comprehension assessed (literal versus inferential) because students with ASD have shown greater difficulty with inferential comprehension processes (Tirado and Saldaña, 2016). In addition to classical predictors, other cognitive factors may help us understand why students with ASD struggle with reading comprehension. Research in typical children has suggested that cognitive flexibility is positively associated with reading (Yeniad et al., 2013). Cognitive flexibility requires the interaction of several mechanisms (attention shifting, conflict monitoring, and perception) that respond to specific environmental demands, such as rule changes, in order to achieve flexible behavior and to solve the problem in a new way (Ionescu, 2012). Precisely, cognitive flexibility is one of the most significant affected executive function in the majority of children and adolescents with high-functioning ASD (Lai et al., 2017). Therefore, it seems logical to expect that they have difficulties in deploying flexible strategies for accomplishing reading tasks.

### Writing Difficulties in ASD

Similar to reading ability, good writing skills are crucial to academic and professional success. In addition, with the growing prevalence of online communication and social networks, the written expression has become a common means of daily interaction between people. Writing represents a major challenge for students with ASD. According to data provided by Mayes and Calhoun (2006), -in a study with children and adolescents with heterogeneous IQ levels-, approximately 60% of students with ASD present some type of specific difficulty in learning to write.

Most of our understanding of the writing process has been based on the recursive and multi-layered model developed by Hayes and Flower (1980) and Hayes (2006), which is composed of three main processes: (1) the *planning process*, directed to prepare the content of the text by retrieving ideas from memory and organizing them; (2) the *translation process*, which includes the grammatical and orthographical encoding, and the motor execution actions involved in handwriting; and (3) the *revision process* that allows writers to compare the written product with their mental representation of the intended text. Research on writing difficulties in ASD has mainly focused on the planning and translation processes. A meta-analysis found significantly worse performance of ASD students on several components of writing, related to the translation process (length, legibility, size, speed, and spelling) and to some indicators of the planning process, such as text structure (Finnegan and Accardo, 2018). Likewise, a more recent study has demonstrated that children with ASD-WID write personal narrative texts that obtain lower ratings in holistic assessments of coherence, structure, and content, and have less lexical and syntactic complexity, in comparison with children with TD (Hilvert et al., 2020). Moreover, fine motor and visuomotor speed problems (Kushki et al., 2011) may often result in illegible or brief writing (Fuentes et al., 2009).

Written expression difficulties of students with ASD have been found not only in narrative discourse, but also in expository and persuasive texts (Brown et al., 2014; Price et al., 2020). In addition, their essays contain more grammatical errors and present less syntactic diversity and complexity, although they can achieve a lexical richness and coherence comparable to their peers with TD (Hilvert et al., 2019).

In summary, variability is the trait that characterizes the academic performance and reading and writing abilities of students with ASD, which is linked to variables, such as intellectual ability, language level, the type of processes involved, and the characteristics of the tasks used to assess reading and writing. In any case, as students progress through the education system, there is a greater emphasis upon reading comprehension and writing expression which gives students access to school curriculum with more autonomy. The transition from primary to secondary school is one important and educational challenge. Research about the experience of young people with autism has identified a number of areas in which they may experience particular concerns compared to their TD peers, including structural/organizational and social demands. Secondary schools tend to be large, with different teachers for different subjects that students need to adapt to Maras and Aveling, (2006). Furthermore, social complexity about forming relationships with a new peer group increases anxieties as well as academic demands of independence (Tobin et al., 2012; Mandy et al., 2016). Our research focuses on the period of adolescence, a stage less studied in the literature, in particular, in the transition from primary to secondary school, an especially demanding challenge for individuals with ASD. Therefore, a measure of transition to secondary education of individuals with ASD without ID was included.

Consequently, the present study seeks to advance the knowledge about the reading and writing performance of students with ASD in several ways. First, there is a paucity of research on studying the relationships between reading and skills and school achievement in individuals with ASD. Second, it is carried out in Spanish, a language with a transparent spelling characterized by a series of phonological and orthographical specificities that can impact psycholinguistic processing. Third, different comprehension processes (literal and inferential) are considered, using, in addition, a reading-specific measure that requires cognitive flexibility. To the best of our knowledge, this measure has not been employed in the previous investigations assessing reading comprehension in ASD. Based on these considerations, the following objectives were addressed as follows:

1. to analyze differences in a wide range of reading and writing skills between adolescents with ASD-WID and adolescents with TD;
2. to analyze differences in academic outcomes in core subjects and on indicators of successful transition to secondary school between adolescents with ASD-WID and adolescents with TD; and
3. to explore the potential contribution of reading and writing skills to academic outcomes in both groups, adolescents with ASD-WID and TD.

According to the literature review, it is expected to find similar results in both groups in reading accuracy and fluency. However, significant lower results are hypothesized in participants with ASD-WID regarding indicators of written expression and reading comprehension tasks tapping inferential and cognitive flexibility processes. Likewise, it is predicted that participants with ASD will obtain lower academic outcomes, as well as worse results in indicators of successful transition to secondary education. Given the prominent role that reading and writing play on school outcomes, it is anticipated that both instrumental skills will have a considerable weight in academic performance, both in the group with TD and in the group with ASD-WID.

## Materials and Methods

### Participants

The present study included 56 adolescents between 12 and 14 years old, of whom 30 were adolescents with ASD-WID (28 males) and 26 were adolescents with typical development (TD; 17 males). The participants had an intellectual functioning within the limits of normality on the K-BIT (Kaufman and Kaufman, 2000).

The group of adolescents with ASD-WID had received a previous clinical diagnosis of an autism spectrum condition by the Psychiatry and Child Neurology services in hospitals and medical centers in the Valencian community at ages ranging between 2 years and 11 months and 6 years old. According to the protocol for the ASD diagnosis, the criteria for ASD from the Diagnostic and Statistical Manual of Mental Disorders fourth edition (DSM-IV; American Psychiatric Association, 1994), the Autism Diagnostic Interview-Revised (ADI-R; Rutter et al., 2006), and/or the Autism Diagnostic Observation Schedule-WPS (ADOS-WPS; Lord et al., 1999) were administered by a multidisciplinary team. In order to confirm the ASD diagnosis for the present study, the Social Communication Questionnaire (Rutter et al., 2003) and the Autism Diagnostic Interview-Revised (ADI-R; Rutter et al., 2006) were administered, taking into account the recommended cutoff points. The results appear in Table 1. These two instruments were administered to the parents by a clinical psychologist from the research team who had been accredited in their application. Likewise, all the adolescents met the strict diagnostic criteria for ASD from the fifth edition of the DSM-5 (American Psychiatric Association, 2013), based on information provided by teachers and parents. Both informants, in interviews with a clinical psychologist, rated the severity of the criteria in the two ASD dimensions on scales ranging from 0 to 3 points (0 represents “almost never,” 1 represents “sometimes,” 2 represents “often,” and 3 represents “many times”).

**Table 1:** Socio-demographic characteristics.

	ASD (*n* = 30)		TD (*n* = 26)			
	M	SD	M	SD	t/F	*P*
Age	12.67	0.80	12.62	0.85	0.05	0.818
IQ	101.83	14.4	101.12	8.77	0.04	0.827
Vocabulary	47.03	7.32	49.88	9.00	1.70	0.197
Parental education	3.46	1.06	3.46	1.07	0.00	0.986
SCQ-Total	14.87	5.67	2.69	1.71	110.7	0.000^*^
ADI-R A	13.45	2.99				
ADI-R B	8.69	2.76				
ADI-R C	5.00	2.40				
	n	%	n	%	*χ*^2^	*P*
Repeat grade in S	7	23.33	1	3.84	4.32	0.038^*^
Educational Support	26	76.66	1	3.84	30.16	0.000^*^
Sex (men)	28	93.33	17	65.38	6.89	0.009^*^

*Parental education measured as highest level of mother or father (0 = elementary school, 1 = Compulsory secondary school, 2 = Medium level vocational training, 3 = Upper secondary education (High School) or Superior level vocational training, and 4 = University degree). Repeat grade in S (Secondary), ADI-R (Autism Diagnostic Interview-Revised), ADI-R A (Qualitative alterations in the reciprocal social interaction), ADI-R B (Qualitative Alterations in Communication), ADI-R C (Restrictive and Stereotyped Behaviors), and SCQ (Social Communication Questionnaire)*.

* *p < 0.05*.

The majority of the adolescents with ASD had educational support at school. Specifically, seven adolescents with ASD (23.33%) were attending school in regular classrooms full time without educational support; nine adolescents (30.00%) attended regular classrooms but received educational support for their specific needs in the high school; and finally, 14 adolescents (46.66%) were placed in the communication and language classroom modality. Furthermore, 11 (36.66%) adolescents with ASD were taking antipsychotic medication (mostly risperidone) for behavioral problems.

The typically developing adolescents had no history of psychopathology or referral to pediatric mental health units (USMI), according to the information found in the school records, and they did not meet DSM-5 criteria for ASD on the screening carried out before beginning the evaluation. None of them were taking any psychoactive medication.

The exclusion criteria for the adolescents who participated in this study were evaluated through an extensive anamnesis carried out with the families. They included neurological or genetic diseases, brain lesions, sensory, auditory, or motor deficits, and an IQ below 80 (see Table 1).

### Measures

The assessment measures were selected based on the objectives of this study. In addition, criteria, such as the psychometric properties of the measures, were taken into account, as well as their translation and adaptation to Spanish.

#### Reading Skills

The assessment of lexical and semantic reading processes was carried out through two tasks from the *PROLEC-SE* battery (*Evaluation of Reading Processes for Secondary Education Students*; Ramos and Cuetos, 1999). Furthermore, a subtest of the *TLC Test* (*Leer para Comprender; Reading for understanding*) was also administered (Abusamra et al., 2010).

##### Lexical Processes Measures

Reading accuracy and reading fluency were assessed through the reading of 40 Spanish words that vary in length, frequency of use, and the complexity of their syllabic structure (i.e., high and low frequency, short and long words). The total accuracy score is obtained by adding up the words read correctly. To obtain the reading speed score, the time spent reading the complete list of 40 words is recorded. In our study reliability index of this measure, Cronbach’s alpha is 0.74.

##### Reading Comprehension Measures

Semantic processes were assessed using one of the tasks proposed in the semantic block of the *PROLEC-SE* battery. The task required silently reading two expository texts: “The Eskimos” and “The Australian Papuans.” After each text is read silently, the text is removed, and the participant has to answer 10 questions. Five questions are literal and can be answered from memory and five questions are inferential; that is, they can only be answered if the participant has understood the text and can make the appropriate inferences. Each correct answer receives 1 point. The total score is obtained by adding up the total number of correct answers on each text.

In this research, we use the direct scores. High direct scores on reading accuracy and comprehension and low scores on reading speed indicate better reading performance. The psychometric properties of the *PROLEC-SE* battery are adequate. For the reliability index, Cronbach’s alpha is 0.84 (Ramos and Cuetos, 1999). In this study, α coefficients for the subscales used were 0.63 (literal comprehension), 0.68 (inferential comprehension), and 0.73 (total comprehension).

Moreover, to assess cognitive flexibility in the reading comprehension process, the “Mental flexibility” subtest from the *Read to Understand Test* (TLC) was administered (Abusamra et al., 2010). In this subtest, instructions are given that involve different ways to approach the reading of a text, and, subsequently, the reader’s awareness of the strategies used is evaluated. Therefore, part of the evaluation is declarative because the student “tells what s/he does” to solve the task. This test assesses, for example, whether the student knows that, in some cases, a superficial understanding of the text is possible (specifically, on tasks, such as searching for five nouns in a paragraph), whereas in other cases, a deeper understanding is necessary. That is, it is not always necessary to pay attention to the meaning of words, sentences, and paragraphs in order to perform certain activities that focus exclusively on grammatical, syntactic, or stylistic aspects, or when searching for a specific piece of information. This test evaluates this type of metacognitive process, which is related to cognitive flexibility, that is, the ability to focus on the reading comprehension process by selecting appropriate strategies. This aspect of metacognition is defined as the ability to cope with a task by selecting the right strategies and modifying cognitive processes in response to changes: to shift attentional focus, select information to guide and choose necessary responses, form plans, and generate monitoring (Abusamra et al., 2010). The test has psychometric properties, such as concurrent validity and internal consistency (Cronbach’s alpha of 0.79). α coefficient for this test in our sample is 0.66.

#### Written Expression

To obtain the spontaneous writing sample, the “Birthday” sheet from the Test of Written Language, fourth edition, was used (TOWL-4; Hammill and Larsen, 2009). The participants were given a blank sheet of paper and a pen and the following instruction: “Write a story about the events and activities shown in the picture.” They were given as much time as necessary to complete the written compositions, which were then transcribed using the Systematic Analysis of Language Transcripts (SALT; Miller and Iglesias, 2010). All the texts were coded on variables that assessed productivity, syntactic complexity, and overall coherence. An independent research assistant was trained in the measures coding during two sessions in a seminar of 5 h. In these sessions, the variables considered were defined and explained and the analysis was practiced on written samples. The research assistant was blind to the goal of the study and did not know the membership group of the texts (ASD or TD). For the reliability check, after the training sessions, the evaluators analyzed approximately 80% of the writings from each group (40 in all), which were randomly selected.

##### Productivity

Children’s productivity, or fluency, was measured as the total number of words in the text. Automated SALT analyses provided information about this variable.

##### Syntactic Complexity

Syntactic complexity was measured as the diversity of complex syntax (Hilvert et al., 2019). This variable was determined by counting the number of different types of complex syntactic devices employed within the text: substantive subordinate clauses, adverbial subordinate clauses, relative subordinate clauses, and coordinated clauses. For the presence of each type of syntactic relationship, 1 point was awarded, with a maximum score of 4. In order to calculate inter-rater reliability, the formula proposed by Sackett (1978) was used (number of agreements divided by the number of agreements plus disagreements, x 100). The agreement was 90% for the substantive subordinate clauses, 100% for the adverbial subordinate clauses, 100% for the relative subordinate clauses, and 100% for the coordinated clauses. All disagreements were resolved by discussion.

##### Global Coherence

To assess the overall coherence of the compositions, the coding system proposed by Barnes and Baron-Cohen (2012) was followed. This system is specially addressed to capture how the story components are included. In this way, it can be identified if there is a cognitive style focused on details at the expense of globality, as the weak central coherence theory states. Thus, the texts were coded on a scale from 0 to 2 for each of the four different elements of the story: (1) setting (where the story takes place); (2) character (who the story is about); (3) conflict (what the story is about); and (4) resolution (how the story ends).

A score of 0 indicates that no information is provided about the element in question; a score of 1 indicates that the composition provides one or more details about the element, but the overall idea of the scene is not captured; and a score of 2 indicates that the narrative provides a well-founded and fully developed view of the corresponding element. With regard to the characters, a score of 0 is awarded if either of the story’s characters is not mentioned, and 1 point if the characters are mentioned, but without defining their relationship to each other or their role in the scene. For conflict, a score of 0 is assigned if the character’s desires or goals are not identified; a score of 1 if a character is defined as wanting something, but without mentioning an obstacle to that goal; and a score of 2 if both a goal and an obstacle to the character achieving that goal are established. Finally, in relation to the resolution component, 0 points are assigned when what happened at the end of the conflict is not mentioned; 1 point if an action that took place at the end of the scene is mentioned; and 2 points if an ending action is described and that action is linked to the overall theme of the scene.

According to the formula proposed by Sackett (1978), the inter-rater reliability of these measures was as follows: in the case of the setting, 100% agreement was reached; for character, 100% agreement; for conflict, 97% agreement; and for resolution, 98% agreement. All disagreements were resolved by discussion.

#### Academic Results

Academic results were reported by parents and/or primary caregivers from the last math, language, and social science evaluation. The numerical range used in the Spanish educational system is from 0 to 10, where 0 is the minimum academic grade and 10 is the maximum.

#### Transition to Secondary Education

Parents filled out a questionnaire derived from a large scale, national transitions study, the Effective Preschool, Primary, and Secondary Education (EPPSE) transitions substudy (Evangelou et al., 2008), used in Makin et al. (2017) to provide an index of “transition success.” Evangelou et al. (2008) defined “successful transition” as a multidimensional construct composed of five underlying factors: developing friendships and confidence, settling into school life, showing a growing interest in school and work, getting used to new routines, and experiencing curriculum continuity.

Parents had to answer six questions related to three of the five factors. Regarding the “developing friendships and confidence” dimension, parents were asked whether, compared to the last year in primary school, their child has more (3 points), the same number (2 points), or fewer (1 point) school friends, as well as more, the same, or less self-esteem, confidence, and motivation. The same scoring system was used in the “experiencing curriculum continuity” dimension, which was assessed by asking parents whether, compared to the last year in primary school, their child shows more, the same, or less interest in school and schoolwork. Regarding the adaptation to school life, parents were asked how they thought their child had settled in (very well, 4 points; quite well, 3 points; not very well, 2 points; and not well at all, 1 point). They were also asked how satisfied they were with the whole process of their child’s transition to secondary school (from very satisfied, 4 points and to not at all satisfied, 1 point), how they felt when their child first moved on to secondary school, and how they feel now (from not at all concerned, 4 points and to very concerned, 1 point). The index of internal consistency Cronbach’s alpha for this questionnaire in our sample is 0.91.

### Procedure

This research was performed in accordance with the ethical standards of the Research Ethics Committee of the University of Valencia, which is regulated by the Ethical Principles for Medical Research Involving Human Subjects (Declaration of Helsinki 1964; World Medical Association General Assembly, 2013). Likewise, the authors received authorization from the Board of Education of the Valencian Government to access the schools and locate the participants.

The evaluation was carried out in the high schools where the adolescents were enrolled, in specially prepared spaces that met optimal conditions for psychoeducational assessment. The informed oral and written consent of the parents of all the participants was also obtained after informing them about the research proposal. The different measures were administered to all the adolescents individually by trained examiners. The parents (mostly mothers) provided information about the adolescent’s transition to secondary education, ASD symptoms, and socio-demographic data.

### Data Analyses

This paper presents a descriptive cross-sectional study of comparison between a group of adolescents with ASD and a group of adolescents with typical development.

The statistical analyses were performed with the statistical program for the social sciences SPSS v 26.0 (SPSS). Preliminary analyses checked all data for multicollinearity and multivariate outliers. The asymmetry and kurtosis data indicate that most of the variables followed a normal distribution (all values between −1 and 1). Variables that did not show a normal distribution were transformed using square-root transformation (coherence setting and coherence character). To compare the reading/writing skill, academic results, and successful transition to secondary school of ASD-WID and TD, Multivariate analysis of covariance (MANCOVA) was used. The data set was examined for violations of essential assumptions associated with the application of MANCOVA. Pearson correlations between all dependent variables pairs and dependent variable-covariate pairs suggested statistically significant linear relationships.

Adolescents’ IQ, vocabulary, and parents’ educational level were included as covariates due to its potential effect on reading and writing abilities and academic outcomes in general. Likewise, the differences between math, language, and social sciences academic outcomes were explored, as well as the differences regarding the transition to secondary education in adolescents with TD and with ASD-WID. Both academic outcomes and parents perceptions about the successful adaptation to secondary education form part of the academic curriculum, which encompasses different interrelated dimensions.

For the ANCOVAs, Bonferroni correction was applied to establish the significance level. The proportion of total variance accounted for by the independent variables was calculated using partial eta squared (according to Cohen (1988): eta squared, 0.06 = small; 0.06–0.14 = medium; and 0.14 = large). To compare academic results and successful transition to secondary school of ASD-WID and TD, *t*-student was used. Moreover, two partial correlations, controlling for parents’ educational level and adolescents’ IQ and vocabulary, were conducted to examine the relationships between reading and writing skills and academic outcomes in both groups. Finally, multiple linear regression analyses were conducted to test the effect of reading and writing skills on the transition to secondary school and academic outcomes in adolescents with ASD-WID.

## Results

### Differences in Reading and Writing Skills Between Adolescents With ASD-WID and Adolescents With TD

The MANCOVA between the ASD-WID and TD groups with the different reading skill scores (accuracy, speed, reading comprehension, and reading comprehension related to mental flexibility), controlling for parents’ educational level, IQ, and vocabulary, was statistically significant [Wilks’ Lambda (Λ) = 0.70, F_6,46_ = 3.21, *p* = 0.01, $\eta_{p}^{2}=0.30$]. Likewise, to calculate the additional ANCOVAs, a significance level of *p* < 0.008 was established, after applying the Bonferroni correction, and the value of $\eta_{p}^{2}$ was calculated to check the strength of the association. Specifically, statistically significant differences were found on the reading comprehension “Mental flexibility” subtest (F_1,51_ = 14.6; *p* < 0.01; $\eta_{p}^{2}=0.22$). In addition, scores for accuracy (word reading), speed, and reading comprehension did not reach the required level of statistical significance between the group with TD and the group with ASD-ID (Table 2).

**Table 2:** Means, standard deviations (SD) of reading and writing skills, and statistically significant differences between ASD and TD adolescents.

	ASD (*n* = 30)		TD (*n* = 26)		*F*_(1, 51)_	*p*	$\eta_{p}^{2}$
	M	SD	M	SD			
*Reading skills*							
P. Reading accuracy	38.07	2.13	39.19	1.87	3.82	0.056	0.07
P. Reading speed	42.60	18.3	33.50	12.3	4.18	0.046	0.07
P. Comprehension	12.80	4.33	14.31	3.95	0.87	0.354	0.01
P. Comprehension L	6.50	2.16	7.54	2.10	2.26	0.139	0.04
P. Comprehension I.	5.90	2.51	6.85	2.05	1.59	0.212	0.03
RC. Flexibility	7.97	2.14	9.73	1.18	14.62	0.000^*^	0.22
*Writing skills*							
Productivity	63.1	44.3	94.2	31.8	7.78	0.007^*^	0.13
Syntactic C	2.13	1.04	3.07	0.74	13.40	0.001^*^	0.21
Coherence S	1.03	0.96	1.15	0.78	0.04	0.826	0.00
Coherence Ch	1.63	0.61	1.96	0.19	4.72	0.034	0.08
Coherence C	1.26	0.94	1.84	0.46	6.51	0.014	0.11
Coherence R	0.90	0.66	1.50	0.76	7.63	0.007^*^	0.13

*P. (PROLEC), P. comprehension (PROLEC reading comprehension), P. Comprehension L. (PROLEC literal comprehension), P. Comprehension I (PROLEC inferential comprehension), RC. Flexibility (Reading Comprehension Mental flexibility), Syntactic C (Syntactic complexity), Coherence S (Setting), Coherence Ch (Character), Coherence C (Conflict), and Coherence R (Resolution)*.

* *p < 0.008 (Bonferroni correction)*.

The MANCOVA between the ASD-WID and TD groups with the different writing skill scores (productivity, syntactic complexity, and coherence), controlling for parents’ educational level, IQ, and vocabulary, was statistically significant [Wilks’ Lambda (Λ) = 0.71, F_6,46_ = 3.02, *p* = 0.014, $\eta_{p}^{2}=0.28$]. Likewise, to calculate the additional ANCOVAs, a significance level of p < 0.008 was established, after applying the Bonferroni correction, and the value of $\eta_{p}^{2}$ was calculated to check the strength of the association. Specifically, statistically significant differences were found in word productivity (F_1,51_ = 7.78; *p* = 0.007; $\eta_{p}^{2}=0.23$), Syntactic complexity (F_1,51_ = 13.4; *p* = 0.001; $\eta_{p}^{2}=0.21$), and Coherence (conflict resolution component; F_1,51_ = 7.63; *p* = 0.007; $\eta_{p}^{2}=0.13$; see Table 2).

### Differences in Academic Outcomes and Transition to Secondary School Between Adolescents With ASD-WID and Adolescents With TD

The MANCOVA between the ASD-WID and TD groups with the different academic outcome scores (language, math, and social sciences) and the transition to the secondary stage was statistically significant [Wilks’ Lambda (Λ) = 0.71, F_6,46_ = 3.02, *p* = 0.014, $\eta_{p}^{2}=0.28$]. Statistically significant differences were found in language (F_1,51_ = 7.78; *p* = 0.007; $\eta_{p}^{2}=0.23$), mathematics (F_1,51_ = 13.4; *p* = 0.001; $\eta_{p}^{2}=0.21$), social sciences (F_1,51_ = 7.63; *p* = 0.007; $\eta_{p}^{2}=0.13$), and the index of successful transition to secondary education (F_1,51_ = 7.63; *p* = 0.007; $\eta_{p}^{2}=0.13$; see Table 3).

**Table 3:** Means, standard deviations (SD) of academic results and successful transition to secondary, and statistically significant differences between ASD and TD adolescents.

	ASD (*n* = 30)		TD (*n* = 26)		F _(1, 51)_	*P*	$\eta_{p}^{2}$
	M	SD	M	SD			
Language	5.96	1.54	7.34	1.59	8.18	0.006^*^	0.13
Mathematics	5.93	1.83	7.61	1.76	9.65	0.003^*^	0.16
Social Science	6.00	1.68	8.07	1.57	18.41	0.000^*^	0.26
Transition to SE	23.70	6.25	27.58	3.76	6.19	0.016^*^	0.11

*Transition to SE (Transition to secondary education)*.

* *p < 0.05*.

### Contribution of Reading and Writing Skills to the Academic Outcomes of Adolescents With ASD-ID and Adolescents With TD

Likewise, two partial correlations were performed, using the covariates of parents’ vocabulary, IQ, and educational level, to analyze the association between the reading and writing variables that showed significant differences between the ASD-WID and TD groups and the learning outcomes in the subjects of the Spanish language, mathematics, and social sciences in the ASD-WID and TD groups.

In the group of children with TD, significant positive correlations were observed between the reading comprehension score related to mental flexibility and the scores on language (*r* = 0.45; *p* = 0.029), mathematics (*r* = 0.56; *p* = 0.006), and social sciences (*r* = 0.40; *p* = 0.050). Similarly, significant positive correlations were observed between the productivity subscale and the scores on language (*r* = 0.51; *p* = 0.013) and mathematics (*r* = 0.44; *p* = 0.035).

In the group of children with ASD-WID, significant positive correlations were observed between the reading comprehension score related to mental flexibility and the scores on language (*r* = 0.37; *p* = 0.050) and mathematics (*r* = 0.37; *p* = 0.050). Likewise, significant positive correlations were observed between the social sciences scores and productivity (*r* = 0.46; *p* = 0.015), syntactic complexity (*r* = 0.61; *p* < 0.001), and coherence (*r* = 0.42; *p* = 0.029; see Table 4).

**Table 4:** Partial correlations between reading/writing skill and academic outcomes.

		Spanish	Mathematics	Social Science
TD	RC. Flexibility	0.455^*^	0.557^*^	0.40^*^
	Productivity	0.511^*^	0.441^*^	0.395
	Syntactic C	0.195	0.133	0.349
	Coherence R	0.259	0.156	0.290
ASD	RC. Flexibility	0.370^*^	0.369^*^	0.064
	Productivity	0.275	0.214	0.462^*^
	Syntactic C	0.335	0.142	0.613^*^
	Coherence R	0.021	0.184	0.420^*^

RC. Flexibility (Reading Comprehension Cognitive flexibility), Syntactic C (Syntactic complexity), and Coherence R (Coherence resolution). ^*^p < 0.05; ^**^p < 0.01. Controlling for IQ, vocabulary, and parental education.

* *p < 0.001*.

Finally, multiple regression analyses were carried out in each group in order to evaluate the contribution of reading comprehension related to mental flexibility and writing skills of productivity, syntactic complexity, and coherence to academic outcomes in the core subjects of language, mathematics, and social sciences (see Table 5). The regressions carried out with the TD group indicated that the predictors that explained the highest percentage of variance in Spanish language were reading comprehension related to mental flexibility, productivity, and coherence, explaining 51% of the total variance. As for the mathematics outcomes, the results showed that reading comprehension related to mental flexibility and productivity were the significant individual predictors, together explaining 55% of the variance. In social sciences, there were no significant individual or collective predictors.

**Table 5:** Multiple regression analysis of reading and writing skills that predict academic outcomes in TD and ASD groups.

	TD			ASD		
	Beta	*t*	*p*	Beta	*t*	*p*
Spanish	*F*_(4,21)_ = 5.64^*^; R^2^ = 51			*F*_(4,25)_ = 2.85^*^; R^2^ = 31		
RC. Flexibility	0.52	3.32	0.003^*^	0.33	2.02	0.05^*^
Productivity	0.38	2.15	0.043^*^	0.17	0.77	0.445
Syntactic C	−0.27	−1.48	0.153	0.27	1.26	0.217
Coherence R	0.36	2.15	0.043^*^	0.02	0.11	0.913
Mathematics	F_(4,21)_ = 6.44^*^; R^2^ = 55			F_(4,25)_ = 2.02; R^2^ = 24		
RC. Flexibility	0.61	4.05	0.001^*^	0.37	2.14	0.042^*^
Productivity	0.38	2.22	0.037^*^	0.14	0.61	0.543
Syntactic C	−0.30	−1.71	0.101	0.08	0.36	0.721
Coherence P	0.29	1.79	0.086	0.15	0.82	0.420
Science	F_(4,21)_ = 2.40; R^2^ = 31			F_(4,25)_ = 8.48^*^; R^2^ = 57		
RC. Flexibility	0.37	1.96	0.063	0.02	0.15	0.879
Productivity	0.17	0.81	0.425	−0.04	−0.26	0.796
Syntactic C	−0.07	−0.33	0.739	0.65	3.78	0.001^*^
Coherence R	0.36	1.81	0.085	0.43	3.07	0.005*^*^

*RC. Flexibility (Reading Comprehension Mental flexibility), Syntactic C (Syntactic complexity), and Coherence R (Coherence resolution)*.

* *p < 0.05*.

The regressions performed with the ASD-WID group indicated that, on the one hand, reading comprehension related to mental flexibility was the single most significant predictor of language and mathematics outcomes, explaining 31 and 24% of the variance, respectively. On the other hand, the predictors that explained the highest percentage of variance in social sciences were syntactic complexity and coherence, explaining 57% of the total variance.

## Discussion

The purpose of this study was to extend the knowledge about academic achievement in adolescents with ASD by addressing various objectives. First, this study analyzed differences in reading and writing performance between adolescents with ASD-WID and adolescents with TD. In terms of reading, no significant differences were found between the two groups on measures of reading fluency (accuracy and speed), which is consistent with most of the research on the topic in languages with opaque orthographies, such as English (Jones et al., 2009).

However, contrary to expectations, our study data failed to show significant differences between participants with ASD-WID and their peers with TD on either literal or inferential reading comprehension. These results contrast with what is commonly reported in the literature, which generally indicates lower reading comprehension competence in individuals with ASD (see meta-analysis by Brown et al., 2013). This discrepancy in the findings could be explained by different factors. Firstly, both groups were matched according to vocabulary knowledge which following the Simple View of Reading Model (Hoover and Gough, 1990) is a fundamental factor for reading comprehension. Numerous investigations with children with ASD have exemplified the strong association between reading comprehension and language (see the meta-analysis by Brown et al., 2013), which emerges even as one of the strongest predictors of the reading comprehension abilities of these students (Davidson et al., 2018). A second important factor is the type of texts employed in our study. We used two expository texts with little social content that do not require the student to display inferential skills related to Theory of Mind, which is clearly affected in ASD (Bora and Pantelis, 2016). In other words, the reader would not have to apply skills that require taking the perspective of the characters, which, in contrast, would be necessary to understand narrative texts (Dore et al., 2018). In fact, studies have shown that individuals with ASD are significantly worse at comprehending highly social texts than less social texts, such as those employed in this study (Brown et al., 2013). Moreover, and in line with our results on inferential comprehension, experimental studies using eye-tracking techniques have shown that adolescents with ASD are capable of developing inferential comprehension skills that are activated automatically (Saldaña and Frith, 2007; Micai et al., 2017), although they may have difficulties on tasks that require them to answer questions that rate this same process (Tirado and Saldaña, 2016). Likewise, another explanation for our discordant results could be found in the composition of the sample, which might consist of students with ASD who have shown strengths in reading performance, both in decoding and comprehension. These profiles have been described in the literature as “skilled readers” (Åsberg et al., 2019) or “average readers” (McIntyre et al., 2017).

However, the results of our study allowed us to identify certain deficient reading comprehension processes in adolescents with ASD, specifically those that require the activation of cognitive flexibility skills and, in particular, metacognitive skills, such as explicitly describing the strategies used when summarizing, detecting the main idea, or giving a title to a text. Thus, significantly lower performance was observed on the reading-specific measure used in this study that requires cognitive flexibility. These results are congruent with the alterations in cognitive flexibility described in ASD. These disturbances have been considered one of its neuropsychological manifestations, although with mixed findings (Leung and Zakzanis, 2014). These outcomes also coincide with the difficulties identified in students with ASD in adapting reading strategies to various reading materials and task demands (Micai et al., 2019). A competent reader must be able to change strategies while reading a text and adapt them to the different objectives and/or requirements presented. Adequate mental flexibility ensures the reader’s active participation in the comprehension process, and its proper functioning facilitates independent learning, which has a great impact on academic performance. Thus, we can see the importance of mastering these processes involved in comprehension and the need to consider this specific area when designing reading intervention programs for individuals with ASD.

In terms of writing skills, as it was hypothesized, the data revealed significant differences between the group with ASD-WID and the group with TD on all the indicators analyzed as: productivity, syntactic diversity, and overall coherence (in the latter case, only in the resolution component). Adolescents with ASD wrote shorter texts with less varied syntactic structures, which is consistent with the results of the previous studies and reviews on the topic (Dockrell et al., 2014; Finnegan and Accardo, 2018; Hilvert et al., 2019). Unexpectedly, both groups performed similarly on most of the variables used to assess overall coherence (setting, character, and conflict). However, significant differences could be identified in the resolution component; that is, the students with ASD failed to mention what happened at the end of the conflict presented in their stories. These difficulties may affect the overall coherence of the written composition, which is consistent with the tenets of the Weak Central Coherence Theory (Happé and Frith, 2006). According to this theory, people with ASD tend to focus on local or marginal aspects of the information and fail to integrate them into meaningful global representations, in this case, by providing an ending related to the conflict narrated. This cognitive style has also been shown in different studies on written expression in ASD, both in children and adolescents (Brown et al., 2014), as well as in the adult population (Barnes and Baron-Cohen, 2012).

The second objective of this study was to analyze the differences in academic outcomes and the transition to secondary school between adolescents with ASD-WID and adolescents with TD. As it was expected, the results showed statistically significant differences in the academic grades reported by parents and teachers in the language arts, mathematics, and social science subjects, and in the families’ perceptions of the transition to high school. These findings reinforce the idea of a significant gap between cognitive ability and school performance, taking into account that the participants in our study did not present intellectual limitations. This means that students with ASD are not displaying their full academic potential, which is being undermined by various factors, including deficits in certain reading comprehension and written performance measures, as discussed below.

Furthermore, according to parents’ perceptions, a less successful transition process to high school and generally worse adaptation were observed in adolescents with ASD compared to their peers with TD. Families report fewer friends and lower self-esteem, confidence, and motivation. They are less satisfied with the transition process and are concerned about it. These results are consistent with the negative experiences described by parents of children with ASD in this period of educational change, referring to social isolation, bullying, and anxiety in their children (Humphrey and Lewis, 2008). Families experience challenges and stress beyond what would typically be expected (Dillon and Underwood, 2012; Mandy et al., 2016; Peters and Brooks, 2016). Therefore, as parents also point out, it is essential for secondary schools to understand the nature of autism and the impact it can have on the child and implement the assistance or support students need for a successful transition, optimal academic performance, and social adjustment (Cremin et al., 2017; Tso and Strnadová, 2017).

Finally, the third objective of this study was to examine the contribution of reading and writing skills to the academic outcomes of adolescents with ASD-WID. As expected, a significant positive relationship was found (in both the ASD-WID and the TD groups) between the two variables being analyzed. Specifically, in the group with ASD-WID, significant positive associations were observed between the reading comprehension measure specifically tapping cognitive flexibility and the grades earned in the language and mathematics subjects. A similar relationship was found between the grades obtained in social sciences and the written expression indicators considered (productivity, syntactic diversity, and coherence). This association highlights the importance of instrumental skills, such as reading and writing, which have a clear influence on school performance. Specifically, on the one hand, our study data indicate that the reading comprehension task involving cognitive flexibility turned out to be the only significant predictor of the grades obtained in language and mathematics. On the other hand, the predictors that explained the highest percentage of social sciences grades were syntactic complexity and coherence.

The difficulties of these students exhibit in certain processes of reading comprehension and written expression have an impact on their academic outcomes, which should lead to designing interventions that fit their profile of strengths and weaknesses. Several research syntheses have supported the use of strategy instruction in the form of question generation, use of graphic organizers, or making predictions (El Zein et al., 2014; Finnegan and Mazin, 2016). More recently, Singh et al. (2020), in a systematic review of case studies, analyzed the effectiveness of a series of instructional procedures that contributed to improving the reading comprehension performance of students with ASD. They highlighted the use of comprehension-enhancing supports in the form of graphic organizers or visual diagrams, metacognitive strategies, collaborative strategies, such as peer tutoring, and computer-assisted instruction. In terms of writing skills, a recent research synthesis carried out by Accardo et al. (2020) identified several effective instructional practices: self-regulated strategy development, sentence frames, video modeling, Handwriting Without Tears®, and analytic task instruction with systematic prompting and graphic organizers. Some of the variables that improved with the application of these techniques were the number of elements in the text structure and the number of words and sentences. It should be noted that, as in the reading comprehension intervention, the studies reviewed were single case studies, and so research using experimental or quasi-experimental group designs is necessary in order to improve the levels of scientific evidence currently available. Apart from these teaching strategies, education professionals may also consider to adjust the assessment criteria or the mode of assessment for adolescents with ASD.

Likewise, parents’ perspectives on the transition process to secondary school suggest the need for tailored assistance at the time of the student’s move to secondary school. In this regard, Peters and Brooks (2016) show that parents describe a more positive transition when both pre-transition support and ongoing support in the learning and social environments of secondary school are implemented. To this end, a better understanding of the difficulties and strengths of students with ASD in this educational stage, their particular style of processing information, and their strengths and weaknesses in reading and writing skills are essential, due to their strong impact on academic performance.

## Limitations

Despite the contributions of these findings, our research has several limitations. First, the relatively small sample size may be hiding some possible significant relationships between the study variables and limiting the possibility to detect small effects. One second limitation, which leads the results to be interpreted with caution, is related to the moderate values of reliability coefficients of the administrated tests. Therefore, future studies should increase the number of participants on a randomized sampling basis and include, apart from psychometric tests, other reading comprehension tasks. The information from criterion-referenced measures could help to design more accurate intervention programs for students with ASD-WID. Third, the mid-range cognitive ability and the gender of the participants with ASD, mostly male, are factors that should be considered, as they may influence the results and affect their generalization to girls or to individuals with ASD with other cognitive levels. In addition, the writing skills of adolescents with ASD were only assessed on a narrative text, and it would have been appropriate to expand the information to persuasive and expository genres. Furthermore, the indicator used to reflect the academic results was the qualification marks of the last evaluation, which may not be representative of the whole academic level of achievement. Finally, our study has a cross-sectional design, and future research should analyze developmental variations in the writing and reading profiles of individuals with ASD.

## Conclusion

This research provides an overview of the school performance of adolescents with ASD and the factors involved in it. The results show that adolescents with ASD have strengths in reading-decoding mechanisms, but their performance on reading comprehension processes involving aspects of mental flexibility and writing skills continues to be significantly below expectations. Both deficits contribute significantly to their academic achievement, which is also below that of their peers with TD and what would be expected based on their intellectual capacity, at a medium range in this study. In any case, academic difficulties should be taken into account when analyzing the parents’ opinions in the transition period to secondary school because parents of students with ASD-WID report a more difficult adaptation process compared to students with TD. Consequently, education professionals face the challenge of adapting their teaching style and employing evidence-based teaching strategies that address students’ different needs while enhancing their capabilities. This may be a promising avenue for reducing the gap between the potential and current academic performance of students with ASD.

## Data Availability Statement

The raw data supporting the conclusions of this article will be made available by the authors, without undue reservation.

## Ethics Statement

The studies involving human participants were reviewed and approved by the University of Valencia. Written informed consent to participate in this study was provided by the participants’ legal guardian/next of kin.

## Author Contributions

IB, AM, BR, and CB contributed to the design of the work and wrote the manuscript. CB was responsible for the analysis and interpretation of data for the study. BR was responsible for the literature search and assessment of the participants. All authors participated in data interpretation and draft the manuscript and approved the version to be submitted.

### Conflict of Interest

The authors declare that the research was conducted in the absence of any commercial or financial relationships that could be construed as a potential conflict of interest.
